# Economic evaluation of an incentive-based program to increase physical activity and reduce sedentary behaviour in middle-aged adults

**DOI:** 10.1186/s12913-022-08294-7

**Published:** 2022-07-19

**Authors:** Jaimie-Lee Maple, Jaithri Ananthapavan, Kylie Ball, Megan Teychenne, Marj Moodie

**Affiliations:** 1 Institute for Health and Sport, Victoria University, Footscray, Australia; 2 Institute for Physical Activity and Nutrition (IPAN), School of Exercise and Nutrition Science, Deakin University, Geelong, Australia; 3 Deakin Health Economics, Faculty of Health, Institute for Health Transformation, Deakin University, Geelong, Australia; 4 Global Obesity Centre (GLOBE), Faculty of Health, Institute of Health Transformation, Deakin University, Geelong, Australia

**Keywords:** Economic evaluation, Cost-effectiveness, Physical Activity, Sedentary Behaviour, Incentivisation

## Abstract

**Background:**

Incentive-based programs represent a promising approach for health insurers to encourage health-promoting behaviours. However, little is known about the value for money of such programs. This study aimed to determine the cost-effectiveness of the ACHIEVE (Active CHoices IncEntiVE) program designed to incentivise increased physical activity and reduced sedentary behaviour in middle-aged adults.

**Methods:**

A within-trial cost-efficacy analysis was conducted. Benefits were assessed by evaluating paired t-tests from participants’ pre- and post- trial Body Mass Index (BMI) (kg/m^2^), sitting time (minutes/day) and metabolic equivalents (METS) minutes. A health sector perspective was adopted for the assessment of costs. Pathway analysis was used to determine the resource use associated with the intervention, with costs expressed in Australian dollars (A$) for the 2015 reference year. A long-term cost-effectiveness analysis was undertaken which extended the analysis time horizon and the trial population to the relevant eligible Australian population. Within this analysis, the 16-week intervention was modelled for roll-out across Australia over a 1-year time horizon targeting people with private health insurance who are insufficiently active and highly sedentary. Improved health related quality of life quantified in Health-Adjusted Life Years (HALYs) (based on the health impacts of increased metabolic equivalent (MET) minutes and reduced body mass index (BMI) and cost-offsets (resulting from reductions in obesity and physical inactivity-related diseases) were tracked until the cohort reached age 100 years or death. A 3% discount rate was used and all outcomes were expressed in 2010 values. Simulation modelling techniques were used to present 95% uncertainty intervals around all outputs.

**Results:**

The within-trial cost-efficacy analysis indicated that the ACHIEVE intervention cost approximately A$77,432. The cost per participant recruited was A$944. The incremental cost-effectiveness ratio (ICER) for MET increase per person per week was A$0.61; minute of sedentary time reduced per participant per day was A$5.15 and BMI unit loss per participant was A$763. The long-term cost effectiveness analysis indicated that if the intervention was scaled-up to all eligible Australians, approximately 265,095 participants would be recruited to the program at an intervention cost of A$107.4 million. Health care cost savings were A$33.4 million. Total HALYs gained were 2,709. The mean ICER was estimated at A$27,297 per HALY gained which is considered cost-effective in the Australian setting.

**Conclusion:**

The study findings suggest that financial incentives to promote physical activity and reduce sedentary behaviour are likely to be cost-effective.

**Trial registration:**

Australian New Zealand Clinical Trials Registry: ACTRN12616000158460 (10/02/2016).

**Supplementary Information:**

The online version contains supplementary material available at 10.1186/s12913-022-08294-7.

## Background

Physical inactivity is a key public health concern in many countries [1]. Currently in Australia more than half (55%) of the adult population do not meet physical activity recommendations, with an estimated 16,000 deaths annually caused by ill health attributable to physical inactivity [2, 3]. A systematic review investigating the economic impact of physical inactivity estimated that the annual healthcare costs ranged from A$681.1 million to A$850 million for the Australian population [4]. In addition, Australian adults engage in sedentary behaviour during 50% to 70% of their waking hours – or 8 to 12 h per day – with the most prevalent activity being watching television [5, 6]. A recent study estimated that sedentary behaviour in Australian adults costs approximately A$185 million in healthcare system costs in one year [7]. A UK-based study estimated the total National Health Service costs attributable to prolonged sedentary behaviour in was £800 million as well as 69,276 avoidable deaths [8]. There is therefore a need for innovative programs to encourage increased physical activity and decreased sedentary behaviour.

The ACHIEVE (Active CHoices IncEntiVE) study is an incentive-based program that aimed to encourage an increase in physical activity and reduction of sedentary time in Australian adults aged 40–65 years. Middle-aged (40–65 years) adults who were insufficiently active and highly sedentary were recruited via a health insurance company to take part in a 16-week feasibility trial. They received incentives (e.g., supermarket vouchers, clothing and cookbooks) for achieving weekly physical activity and reduced sitting time goals, calculated using their baseline behaviour. The program also involved a motivational interview, weekly text messages and Fitbits distributed to participants to monitor their progress. As a result of the intervention, participants’ mean leisure-time physical activity increased by 252 min/week; mean transport-related physical activity increased by 178.5 min/week; and mean sitting time decreased by 3.1 h/day (all *p* < 0.001) [9]. These changes were assessed by the International Physical Activity Questionnaire Long version (IPAQ-L) pre- and post- intervention analyses, which has been established as an acceptable measurement of both physical activity [10] and sedentary behaviour [11]. Only leisure-time and transport-related physical activity was reported upon in the outcome paper [9]. This was due to the other physical activity domains (e.g., work-related and domestic-related) being much less discretionary (i.e., people have much less choice/control over them). Furthermore, body mass index (BMI) and systolic blood pressure decreased significantly in both men and women, whilst diastolic blood pressure decreased in men [9].

The ACHIEVE program demonstrated promising results using an incentive strategy to target both physical inactivity and sedentary behaviour, however in order to determine the value for money of the program, it is essential that its economic credentials are also assessed. Although the effectiveness and cost-effectiveness of physical activity interventions and policies have been well explored [12], to date only two studies have assessed the cost-effectiveness of incentive strategies to increase physical activity in adults [13, 14] and both showed potential for cost-effectiveness. The role of sedentary behaviour is less investigated and to our knowledge, no studies have assessed the cost-effectiveness of using incentives specifically to reduce sedentary behaviour. The aim of this study is to examine the economic credentials of the ACHIEVE program, by assessing its short term cost-efficacy in a within-trial analysis, and its potential for cost-effectiveness by modelling the long term health benefits and health care cost-savings resulting from improved physical activity levels and reduced BMI.

## Methods

### Overview

The study draws on the efficacy data from the ACHIEVE study conducted in 2015 [9]. Both a within-trial cost-efficacy analysis and an evaluation reporting the potential cost-effectiveness if the intervention was scaled up and rolled out to all eligible participants throughout Australia, have been undertaken. Results of the cost-efficacy analysis are reported as incremental cost-effectiveness ratios (ICER) calculated as cost (A$) per metabolic equivalents (METS) increased, sitting time reduced, and BMI unit reduced. The cost-effectiveness analysis reports ICERs as the cost per health adjusted life year (HALYs) gained.

### The ACHIEVE study

Details of the ACHIEVE study have been previously published [9] and protocol information can be accessed on the Australian and New Zealand Clinical Trials Registry: ACTRN12616000158460 (10/02/2016). Key features of the study relevant to the economic evaluation are described here.

#### Recruitment

Participants were recruited to the ACHIEVE study through Geelong Medical and Hospital Benefits Association (GMHBA), a not-for-profit health insurance fund in Victoria, Australia. Study recruitment was facilitated through invitations to participate distributed via e-mail to potentially eligible members (*n* = 1,544) based on GMHBA client database information. The study was targeted at adults aged 40–65 years, as this is the life stage characterised by declining levels of physical activity and increased risk of chronic disease onset [6, 15]. Eligibility criteria was determined by participant self-report and included living within 25 km of the study site (for pragmatic reasons), not meeting current physical activity guidelines (i.e. undertaking less than 150 min/week of moderate-vigorous physical activity) and spending more than three quarters of the day sitting on most days of the week [16]. A total of 36 men and 46 women were recruited to the program.

#### The intervention

Over a 16-week period, participants were encouraged to increase their physical activity to 150 min/week and reduce their sitting time by 150 min/week in progressive increments. Activity was monitored for incentive distribution by Fitbit devices that participants retained at the conclusion of the program. Participants were informed that regular syncs via their mobile or computer were essential in order to upload their data to the ACHIEVE project website. Participants were also required to place their device on ‘sleep mode’ each night to ensure only sedentary time whilst awake was recorded. A point-based incentive scheme was administered with participants receiving one point per minute of physical activity increased (capped at 30 min per day) and one point per minute of sitting time reduced in comparison to their baseline measures. Weekly physical activity behavioural goals included achieving 100 min/week (for the first four weeks); 120 min/week (month 2); and 150 min/week (month 3 & 4). Weekly sitting time goals included achieving a 100 min reduction/week (for the first four weeks); 120 min reduction/week (month 2); and 150 min reduction/week (month 3 & 4). If goals were met (and sufficient points were accrued), participants received the corresponding weekly reward. Rewards ranged in value from A$7 to A$50 and included clothing, supermarket vouchers and cookbooks. A lottery schedule incentive was also offered in the final week that gave eligible participants a chance to win one of four iPad mini devices (valued at approximately A$450). The main incentive component was supplemented by additional support through a motivational interview at intervention commencement plus weekly motivational text messages (*n* = 16). Text message content aimed to encourage participants and provide strategies to increase physical activity and reduce sedentary behaviour.

#### Measures

A pre-post intervention design was employed with measurements at baseline and post-intervention (16-weeks). Physical activity and sedentary behaviour were measured using the IPAQ-L, which is a 27-item self-report measure that assesses duration and frequency of physical activity in the last 7 days [17]. The domains include job-related, transport, domestic and leisure-time physical activity as well as time spent sitting. Categories are also broken down into walking (for 10 min or more) and cycling for transport, and moderate-intensity and vigorous intensity for leisure time physical activity.

BMI (kg/m^2^) was calculated from height (objectively measured by researchers at baseline) and weight (objectively measured by researchers at baseline and by participants post-intervention using Wi-Fi scales provided by researchers and retained by participants).

For the purpose of this economic evaluation, the main outcome measures of interest were the mean differences between baseline and post-intervention (16 weeks) IPAQ-L scores for leisure-time and transport physical activity, sitting time, and BMI.

### Within-trial cost-efficacy analysis

#### Assessments of benefits

Results from the paired t-tests for changes from baseline in BMI (kg/m^2^) and sitting time (minutes/day) as reported in the ACHIEVE outcomes paper were used in this analysis [9]. In addition, participant physical activity reported in the IPAQ-L questionnaire was used to calculate MET minutes per person per week. This was scored by multiplying the MET intensity assigned to the activity by the time (minutes) spent partaking in this activity and again by the number of days this activity was undertaken [18]. In accordance with the protocol for scoring the IPAQ-L [17], one extreme outlier of leisure time physical activity was truncated to 21 h (i.e., 3 h/day). STATA was used to run paired t-tests for pre-post METS.

#### Assessment of costs

A health sector perspective was adopted for the economic evaluation. Pathway analysis was conducted to identify component activities and associated resource utilisation and costs entailed in the implementation and monitoring of the ACHIEVE intervention. Records kept by the project manager were used to ascertain cost components and unit costs. Cost items included website design, participant recruitment, program administration, motivational text messages, website monitoring, incentives, and postage. Research costs associated with the intervention (e.g., the project manager’s time spent recruiting research assistants, working on ethics applications, and outcome measurement) were excluded in the base case analysis, but were included in the scenario analysis. Where the project manager’s records did not include the required details for the costing, unit cost estimates were made using credible sources such as Australian Bureau of Statistics average weekly earnings [19] and variability around these estimates were incorporated in the uncertainty analysis. The reference year for the within trial cost-efficacy analysis is 2015, the year that the ACHIEVE study was undertaken.

Results of the within-trial analysis were calculated as the cost per BMI unit lost per participant, cost per MET increase per participant per week and the cost per minute of sedentary time reduced per participant per day. Given that there are no willingness to pay thresholds for these intermediate outcomes measured in ACHIEVE, an assessment of cost-effectiveness cannot be made. It is therefore up to decision-makers to assess whether the incremental cost-effectiveness ratios for the intermediate outcomes represent good value for money.

The ACHIEVE within-trial cost components are reported in Additional file 1: Appendix 1.

### Scaled-up cost-effectiveness analysis

#### Recruitment

A long-term cost-effectiveness analysis was undertaken which extended the analysis population to the relevant Australian population and the time horizon to over the lifetime of the eligible population (until death or age 100 years). The intervention was assumed to be operating in ‘steady state’ (i.e., running at its full effectiveness potential) and was measured against a ‘do nothing’ comparator. The intervention timeframe remained 16-weeks (as in the initial ACHIEVE trial) and was delivered to eligible participants over the course of one year. Eight private health insurers were identified to deliver the incentive program, representing approximately 93% of the Australian private health insurer market share [20]. Eligibility for service provider inclusion was having equal or higher health insurance market share than the original trial insurer (GMHBA, who has approximately 2.1% of the market share) [20].

The eligible population represented the 2010 Australian population aged 40–65 years adjusted to include those with private health insurance [21] (approximately 56% of the population) who were insufficiently active (approximately 58%) based on the 2011–12 Australian Health Survey [6]. The uptake of the scaled up intervention was informed by uptake of the ACHIEVE study (approximately 12%) [9]. The impact of a higher uptake rate was tested in the sensitivity analyses. This was informed by uptake rates in a similar study [22] (sedentary adult population who were provided with step count goals and used the IPAQ-L for outcome measurement) which reported an uptake rate of 37%. However, due to the age restrictions of this study (40–65 year olds) a lower uptake rate of 30% was used (Table 1).

**Table 1:** Sensitivity analyses

Scenario	Description
Scenario 1	Within-trial analysis including research costs
Scenario 2	Scale up analysis where the intervention effect was assumed to be maintained over the lifetime of the population
Scenario 3	Scale-up analysis where the intervention effect was assumed to be maintained for one year
Scenario 4	The Scale-up base case analysis used average staff costs for ‘Financial and insurance services’. This scenario assumed a lower wage rate using the average salary for ‘Administrative and Support Services’ [19]
Scenario 5	Uptake rate was assumed to be 30%. This was informed by a study with similar study design which had an uptake rate of 37% [22], adjusted to reflect the age restrictions in the ACHIEVE study

#### Benefit analysis

The change in METS and BMI as a result of the intervention were used to estimate the long-term health impact of the intervention compared to a ‘do nothing’ comparator. A previously developed and validated multi-state life table Markov model—The ACE-Obesity Policy model—was used in the analysis [23]. Details of the model have been previously published and a brief description follows [23, 24]. The model simulates the effects of the intervention-related changes to the distribution of BMI and physical activity levels (measured in METS) in the intervention population on the incidence of nine diseases causally related to BMI (breast cancer, colorectal cancer, endometrial cancer, kidney cancer, type 2 diabetes, hypertensive heart disease, ischaemic heart disease, stroke and osteoarthritis of the hip and knee), and five diseases causally related to physical inactivity (breast cancer, colorectal cancer, type 2 diabetes, ischaemic heart disease and stroke) [23]. Reduced incidence of diseases resulted in reductions in the prevalence and disease-related mortality and morbidity, thereby improving long term health outcomes (reported as HALYs) and producing healthcare cost-savings [23, 24].

The short-term impact of the intervention was assessed over the 16-week intervention period. There was no maintenance measurement in the ACHIEVE study and therefore it is unknown how long the intervention effects were maintained. In the base case scale up analysis it was assumed that there would be no intervention effect remaining after five years. Due to the lack of current literature on maintenance effects once incentives are removed, this assumption was informed by a meta-analysis that found that participants in diet and exercise programs keep weight off for an average of 6 months but then commence regaining weight at a rate of 0.03 BMI-points per month until, at around 5.5 years post-intervention, no effect remains [25]. Variations in this assumption with intervention effect being maintained over the lifetime and for one year were tested in sensitivity analyses (Table 1).

#### Cost analysis

Modifications were made to the costing of the intervention in the ACHIEVE trial to enhance the feasibility of scale-up and to reflect the intervention’s likely implementation under non-research conditions. It was assumed home-visits for initial baseline measurement would not be undertaken as this is a research related activity and therefore travel costs were excluded. It was assumed that Fitbits would be distributed via post and these costs were included in the scale up analysis. The wifi-scales that were distributed in the ACHIEVE study were excluded in the scale up analysis as they are only required to measure the outcome of the study. Personnel costs included the cost of website development as in the ACHIEVE study, a cost assumed to accrue to each of the health insurers. In the base case scale up analysis, it was assumed that each of the insurers would require two full time staff to recruit and administer the program. The impact of lower staff wage rates was tested in the sensitivity analyses. Additional hourly staff costs for the preparation and mail out of the incentives were also included (assuming 10 incentives could be prepared and mailed out each hour). The cost of the incentives and the number of incentives per participant were taken from the ACHIEVE study.

To maintain consistency with the inputs of the ACE-Obesity Policy model, the analysis was undertaken for the 2010 cost year. Costs taken from the ACHIEVE study were adjusted to 2010 values using the gross domestic product (GDP) price index reported by the Australian Institute for Health and Welfare [26]. All costs and benefits were measured over a lifetime time horizon (up to 100 years or death) and were discounted at a 3% annual rate [27].

#### Sensitivity analyses

Sensitivity analyses were undertaken to assess the impact of key variables or assumptions on the ICER results. Analyses were undertaken with varying assumptions related to the duration of intervention effect, staff wage rates and the intervention uptake rate (Table 1).

#### Uncertainty analysis

Resource use for several cost items from the ACHIEVE study was estimated retrospectively by the project administrator, therefore variability of ± 20% in the values was incorporated using a Pert distribution [28]. Monte-Carlo simulation using the add-in tool Ersatz (EpiGear, Version 1.35) [28] was used to undertake uncertainty analyses to test the robustness of the results taking into consideration the variability around model input parameters. All results are presented with 95% uncertainty intervals (the range within which the true value lies with 95% certainty). Two thousand iterations of the model were conducted; for each iteration, values were randomly chosen from the specified distribution for each input variable (Additional file 1: Appendix 1).

#### Assessment of cost-effectiveness

Whilst a willingness to pay (WTP) threshold is not explicit in Australia, a commonly used threshold to determine value for money in the Australian context is A$50,000 per HALY gained [24, 29–31]. The intervention was considered cost-effective if the resulting ICERs were below this threshold.

## Results

### Within-trial cost-efficacy analysis

The total ACHIEVE intervention cost approximately A$77,432 and A$110,644 when research costs were included. The base case cost per participant recruited was A$944. The ICER per BMI unit lost per participant was A$763; MET reduction per participant per week was A$0.61 and minute of sedentary time reduced per participant per day was A$5.15. ICER results when research costs were included are shown in Table 2.

**Table 2:** Within-trial analysis results

	Total intervention cost (A$2015)	Cost per person (A$2015)	ICER per BMI unit loss per person (A$2015)	ICER per MET increase per person per week (A$2015)	ICER per reduction in sitting time (minutes) per person per day (A$2015)
Within-trial analysis base case	77432 (71289; 83628)	944 (869; 1020)	763 (607; 946)	0.61 (0.45; 0.82)	5.15 (4.12; 6.31)
Within-trial analysis scenario 1 – including research costs	110644 (105743; 115468)	1349 (1290; 1408)	1090 (878; 1329)	0.87 (0.64; 1.16)	7.36 (5.98; 8.97)

### Scale up cost-effectiveness analyses

**Fig. 1:**
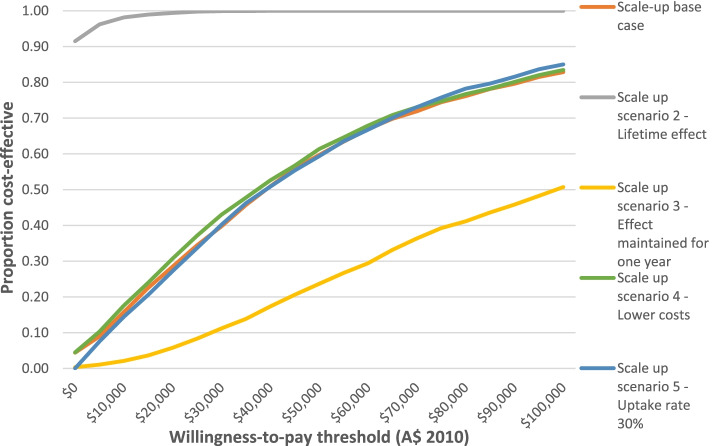
Cost-effectiveness acceptability curve. Note: A$; Australian Dollar. The y-axis represents the proportion of each scenario being cost-effective at any given willingness-to-pay threshold; the x-axis represents the corresponding willingness-to-pay thresholds

**Fig. 2:**
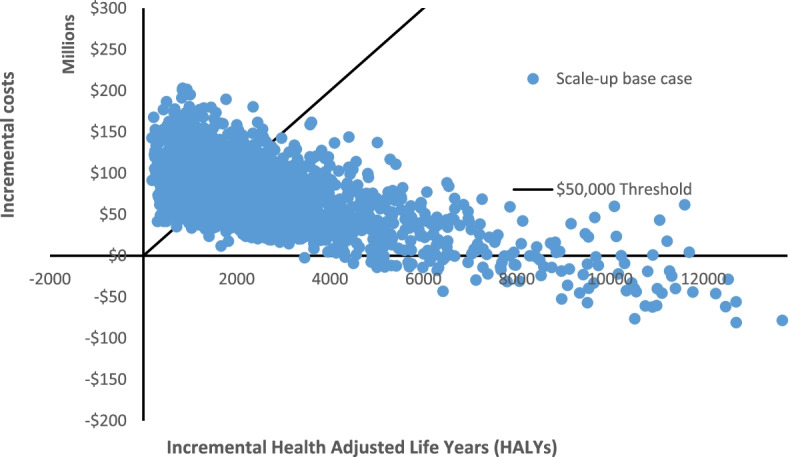
Scale up base case cost-effectiveness plane. Note: HALYs; Health-Adjusted Life Years. The y-axis represents the incremental costs of the scale up base case scenario; the x-axis represents the incremental HALYs gained. Approximately 60% of the iterations modelled for the scale up base case fell below the cost-effective threshold

If the incentive program was rolled out nationally, a total of 131,623 males and 133,472 females were estimated to be eligible (approximately 3.7% of Australians aged 40–65 years in 2010 [21]). The scale up base case and all scale up scenarios modelled were found to be cost-effective (ICER less than A$50,000 per HALY gained), with the exception of scale up scenario 3, where the effect was assume to be only maintained for one year (Table 3 and Fig. 1 below). Approximately 60% of iterations modelled for the scale up base case fell below the cost-effectiveness threshold (Fig. 2). Scale up scenario 2 demonstrated that if the intervention effects were maintained over the lifetime then the program would be dominant, indicating the intervention it is both cost-saving and health promoting.

**Table 3:** Scale-up cost-effectiveness results

	**Total intervention cost (A$2010)**	**Health care cost savings (cost offsets, A$2010)**	**Total HALYs gained**	**Total ICER**	**Proportion of iterations that were cost-effective (< A$50,000 per HALY gained)**
Scale-up base case	107355577 (56075749; 174774252)	33399577 (5581663; 115828487)	2709 (453; 9518)	27297 (dominant; 234905)	60%
Scenario 2 – Lifetime effect	107110520 (56712591; 172073021)	388571651 (66607772; 1310538331)	31830 (5419; 108,338)	dominant (dominant; 7322)	100%
Scenario 3—Effect maintained for 1 year	105771678 (55934827; 169,479,941)	14862624 (48448335; 2631784)	1217 (215; 3884)	74683 (12054; 520362)	24%
Scenario 4 – Lower costs	103707187 (54356276; 167050251)	33566209 (5759678; 113317956)	2725 (459; 9544)	25742 (dominant; 221992)	61%
Scenario 5 – 30% uptake	261364128 (207614656; 321623049)	82032666 (14128156; 263626923)	6685 (1108; 21,152)	26827 (dominant; 222625)	59%

Note: Values are mean (95% confidence interval); dominant: the intervention is both cost-saving and improves health

## Discussion

The ACHIEVE intervention cost per participant recruited was A$944. The ICER for MET increase per person per week was A$0.61; minute of sedentary time reduced per participant per day was A$5.15 and BMI unit lost per participant was A$763. The ACHIEVE program showed the potential to be cost-effective if scaled up across the country. The long-term cost-effectiveness analysis showed that if the ACHIEVE program was scaled up to all eligible individuals across Australia, 265,095 adults aged 40 to 65 years would be recruited and the program would have the potential to be cost-effective (ICER: A$27,297). However, sensitivity analyses demonstrated that if program benefits were only maintained for one year, the intervention would not be cost-effective. To address this uncertainty, future research should prioritise collecting long-term data to estimate the longer term effectiveness and cost-effective of incentive based behaviour change programs. Other sensitivity analyses (lower staff costs, increased uptake rate) produced similar mean ICERs to the base case and were all below the cost-effectiveness threshold. Due to the high variability in effectiveness and the costs of the scaled up intervention, between 24–61% of the iterations modelled were predicted to be cost-effective. These analyses suggest that ACHIEVE and similar incentive-based programs to increase physical activity and reduce sitting time are likely to represent good value for money if the intervention effect lasts longer than one year.

Only two previous studies have evaluated the cost-effectiveness of incentive-based programs for increasing physical activity. Participants in those studies included employees in workplaces in Ireland [13] and physically inactive members of the public in a London borough [14]. Incentives used in those studies included various products from local businesses [13] and free leisure centre memberships [14]. Physical activity was monitored by self-report point systems for physical activity minutes [13] and attendance at local leisure centres [14]. Costs were assessed from both healthcare provider [13, 14] and an employer perspective [13]. Both of these studies demonstrated potential for cost-effectiveness; however, the results were limited by wide confidence intervals [13] and a lack of assurance around sustainability of benefits [14]. Similar issues were observed in the current study. Confidence intervals of modelled scenarios were wide and in most instances crossing the threshold of cost-effectiveness (ICER > 50,000).

The provision of membership rewards by health insurance providers is becoming more common in Australia and internationally. Traditionally, these incentives were a marketing strategy offered to increase the appeal of initiating membership by providing discounted access to health-related products and services such as gym memberships, Fitbits and exercise equipment. However, in recent years, private health insurance providers have increasingly rewarded members for *maintaining* a healthy lifestyle [32–34]. Although these programs intuitively appear beneficial from a public health stance, it is essential that they are evaluated on their economic credentials to inform resource allocation and service design. An intervention based on the notion of encouraging maintenance of a healthy lifestyle was modelled in the ACE-obesity policy study, which assessed financial incentives for weight loss by private health insurers [35]. The intervention targeted adults (18 +) who were overweight or obese and had extras/ancillary cover. Members received financial incentives from their health insurance if weight loss/maintenance goals were met ($200 cash payment per year). This was offered alongside an initial one-year weight loss program. Results from that study indicated good value for money from a societal perspective [35]. However, it was not found to produce a positive return on investment to the private health insurer.

### Limitations

The methods used in the initial ACHIEVE feasibility trial restricted the scope and robustness of this economic evaluation. Firstly, given the lack of a control group, it is difficult to assess whether the effectiveness of the intervention is specific to certain characteristics of those who chose to participate and how well the effectiveness is generalizable to the whole eligible population. The procedure for collecting demographic information in recruitment screening did not allow for this information to be linked with outcome data. Having more individualised data would have provided insight into the differential engagement and appeal of this type of intervention based on population characteristics. In addition, the measurement of the two primary behavioural outcomes was via self-report. Although the IPAQ-L is widely used as a measure of physical activity and sitting time, data collected via the IPAQ-L can be subject to recall difficulties and bias, and is susceptible to over-reporting of physical activity and under-reporting of sedentary time [36]. However, main outcomes also report improvements in measured BMI and blood pressure consistent with these self-report physical activity changes. Another limitation impacting the economic evaluation was that health-related quality and healthcare resource use were not collected in the ACHIEVE study.

Another limitation was the sample of the initial ACHIEVE trial. Although GMHBA has a membership that is socio-demographically diverse [9], it is highly likely that individuals of a low socioeconomic position were underrepresented. An alternative would be to consider providing such programs as part of the publicly funded Medicare system in Australia. Improving health equity by broadening the scope of these programs beyond individuals within the population who hold private health insurance would undoubtedly increase the societal benefits, but also the costs of the intervention [35]. Therefore, exploring this approach in varying population groups should be a focus of future programs and corresponding economic evaluations.

Another limitation of this study is the analyses were undertaken from the health sector perspective. Physical activity and sedentary behaviour interventions have important societal implications (e.g. productivity impacts) which were not included in the analysis. It is therefore recommended that future studies incorporate a broader perspective to capture the societal impacts of the intervention.

The study is also limited by the lack of literature examining the maintenance effects of incentive-based programs. Currently there is limited evidence on the sustained effects of incentive-based physical activity or sedentary behaviour programs once incentives are removed. As BMI was an outcome variable in the initial trial, the use a meta-analysis which examined weight loss maintenance resulting from weight loss programs [25] was considered appropriate to guide our base case model assumptions. However, the sensitivity analyses demonstrated that the results of the cost-effectiveness analysis were highly dependent on assumptions related to how long the intervention effects were maintained.

There were also limitations related to the ACE-Obesity Policy model’s ability to fully capture the benefits of the ACHIEVE study. The model was unable to estimate the health benefits of reduced sitting time and therefore these benefits were not included in cost-effectiveness analyses. Future model developments should focus on incorporating sedentary behaviour as an independent risk factor for long term chronic disease to allow better estimation of the economic credentials of interventions that target this important risk factor.

### Strengths

Despite these limitations, a key strength of the current study is that we were able to supplement the within-trial analysis results with modelled long-term results which can estimate whether the ACHIEVE study represents good value for money. In addition, the modelling was based on a pilot study led by a health insurer in a real-life Australian setting.

To our knowledge no economic evaluations of incentive programs to reduce sedentary behaviour have been conducted and therefore our within-trial results represent an important contribution to the literature.

## Conclusions

This study outlined both the economic credentials of the ACHIEVE study and modelled the program to all eligible Australians to highlight the potential long-term cost-effectiveness of this incentive program. Incentives are often used simply as a marketing strategy, however there is real potential for their use as a cost-effective health promoting intervention. Potential challenges for future programs may include the ability to design programs that encourage maintenance beyond the duration of the intervention and ensuring uptake and investment in these programs by health insurance providers (due to concerns of positive returns on investment). Future research should aim to collect long-term effectiveness data to improve accuracy of cost-effectiveness evaluations to inform resource allocation decisions. Exploring this approach in varying population groups should also be considered in future programs and economic evaluations.

## Supplementary Information

**Additional file 1: Appendix 1.** ACHIEVE program component costs (A$2015).

## Data Availability

The datasets used during the current study are available from the authors on reasonable request.

## Abbreviations

ACHIEVE: Active CHoices IncEntiVE study
A$: Australian Dollar
BMI: Body Mass Index
IPAQ-L: International Physical Activity Questionnaire Long version
METS: Metabolic Equivalents
ICER: Incremental Cost-Effectiveness Ratio
HALYs: Health-Adjusted Life Years
GMHBA: Geelong Medical and Hospital Benefits Association
GDP: Gross Domestic Product
IT: Information Technology
WTP: Willingness To Pay
