# Supplementary material for: Economic evaluation of an incentive-based program to increase physical activity and reduce sedentary behaviour in middle-aged adults
Source: BMC Health Serv Res. 2022 Jul 19;22:932. doi: 10.1186/s12913-022-08294-7 (PMC9297637; doi:10.1186/s12913-022-08294-7)
Supplement: Supplementary file 1 — Additional file 1: Appendix 1. ACHIEVE program component costs (A$2015). [file 12913_2022_8294_MOESM1_ESM.docx]

**Appendix 1. ACHIEVE program component costs (A$2015)**

| Program component | Resource item | Resources use (hours) | Resource use (proportion of time) | Uncertainty distribution and range used (Pert+/-20%) | Unit (km) | Unit cost | Source |
| --- | --- | --- | --- | --- | --- | --- | --- |
| Website development | Deakin IT staff | 204 |  | 6972.4;  10,459 |  | 43.86 | Project Manager records |
| Recruitment Screening | GMHBA staff | 3 |  | 2.4; 3.6 |  | 37.25 | Australian Bureau of Statistics, *Average Weekly Earnings, Australia, May 2015*. 2015, ABS: Canberra. [[16](#_ENREF_16)]  Project Manager records |
| Project administration (Manager) | Deakin Project Manager |  |  |  |  | 34,467 | Project Manager records with on costs included |
|  |  |  |  |  |  |  |  |
| Program administration (RA) | Deakin RA casual staff |  |  |  |  | 30,376 | Project Manager records with on costs included |
| Travel to baseline visit | Deakin RA casual staff |  |  | 5; 25 | 450 | 22.13 | Transport and Infrastructure Council. *Australian Transport Assessment and Planning Guidelines*. 2016 [cited 1 May 2017]; Available from: https://www.atap.gov.au/parameter-values/road-transport/2-vehicle-operating-cost-voc-components |
| Measurement tool | Wi-Fi scales | 92 |  |  |  | 46 |  |
| Website monitoring | Deakin IT staff | 16 |  | 12.8; 19.2 |  | 43.86 | Project Manager records |
| Incentive monitoring tool | Fitbit One |  | 98 |  |  | 129 | Project Manager records |
| Motivational SMS | Telstra |  |  |  |  | 75 | Telstra. *Telstra Desktop Messaging*. 2012. https://www.telstra.com.au/content/dam/tcom/business-enterprise/mobility-solutions/Mobile%20Messaging/pdf/telstra-desktop-messaging-brochure.pdf. Accessed from 14 June 2016. |
| Achieved PA incentive 1  (type 1) | Scarf |  | 26 |  |  | 7.5 | Project Manager records |
| Achieved PA incentive 1  (type 2) | Cap |  | 28 |  |  | 10.50 | Project Manager records |
| Achieved PA incentive 2 | Supermarket voucher |  | 50 |  |  | 10 | Project Manager records |
| Achieved PA incentive 3 | Cookbooks |  | 34 |  |  | 18.50 | Project Manager records |
| Achieved PA incentive 4 | Supermarket voucher |  | 33 |  |  | 50 | Project Manager records |
| Achieved SB incentive 1 | Supermarket voucher |  | 35 |  |  | 10 | Project Manager records |
| Achieved SB incentive 2 | Shirt |  | 27 |  |  | 18 | Project Manager records |
| Achieved SB incentive 3 | Supermarket voucher |  | 21 |  |  | 40 | Project Manager records |
| Achieved SB incentive 4 | Hoodie |  | 20 |  |  | 38 | Project Manager records |
| Draw incentive 5 | Ipad |  | 4 |  |  | 453.64 | Project Manager records |
| Incentive postage – Ipad | Australian Post satchels |  | 4 |  |  | 8.13 | Project Manager records |
| Incentive distribution - all other incentives | Australian Post satchels |  | 274 |  |  | 4.76 | Project Manager records |

*All costs are in Australian dollars (A$)
*Abbreviations*: *ABS* Australian Bureau of Statistics, *FTE* full-time equivalent, *PA* physical activity, *SB* sedentary behaviour, *SMS* short message service, *RA* research assistant, *GMHBA* Geelong Medical and Hospital Benefits, *IT* information technology
